# ISG-15, beyond its functions in the cell: a mini review

**DOI:** 10.1007/s00018-025-05705-w

**Published:** 2025-07-28

**Authors:** Mudassir S. Ali, Yun Sang Tang, Horace Hok Yeung Lee, Susan C. Baker, Chris Ka Pun Mok

**Affiliations:** 1 Department of Microbiology and Immunology, Stritch School of Medicine, Loyola University, Chicago, Maywood, IL 60153 USA; 2 Infectious Disease and Immunology Research Institute, Stritch School of Medicine, Loyola University Chicago, Maywood, IL 60153 USA; 3 The Jockey Club School of Public Health and Primary Care, Faculty of Medicine, The Chinese University of Hong Kong, Hong Kong SAR, P.R. China; 4 Li Ka Shing Institute of Health Sciences, Faculty of Medicine, The Chinese University of Hong Kong, Hong Kong SAR, P.R. China; 5 Centre for Regenerative Medicine and Health, Hong Kong Institute of Science & Innovation, Chinese Academy of Sciences, Beijing, China; 6 SH Ho Research Centre for Infectious Diseases, Faculty of Medicine, The Chinese University of Hong Kong, Hong Kong SAR, China; 7 School of Biomedical Sciences, Faculty of Medicine, The Chinese University of Hong Kong, Hong Kong SAR, China

**Keywords:** ISG-15, Extracellular, Interferon, LFA-1, Innate immunity

## Abstract

Interferon-stimulated gene 15 (ISG15) is an interferon-stimulated gene and a ubiquitin-like protein, traditionally known for its role in ISGylation. In addition to its intracellular functions, recent studies have revealed a novel role for extracellular ISG15, particularly in the context of viral infections. Beyond type I interferons, various stimuli, including viral and bacterial infections, have been found to trigger its secretion. Notably, the integrin receptor LFA-1 has been identified as a receptor for extracellular ISG15. Despite these advancements, the precise mechanisms by which extracellular ISG15 functions—such as the pathways regulating its secretion and receptor interactions—remain unclear. Viral proteins and de-ISGylating enzymes are known to influence ISG15 secretion levels, thereby impacting its immunomodulatory potential. This mini-review summarizes the existing studies aimed at understanding the mechanisms behind the secretion and functions of extracellular ISG15, with a particular focus on its immunomodulatory effects during infection. We also explore the contrasting roles of extracellular ISG15 in mice and humans, highlighting the need for more species-specific research. Further investigation into the role of extracellular ISG15 may uncover novel therapeutic strategies for infectious diseases, cancer, and inflammatory conditions.

## Introduction

**Fig. 1:**
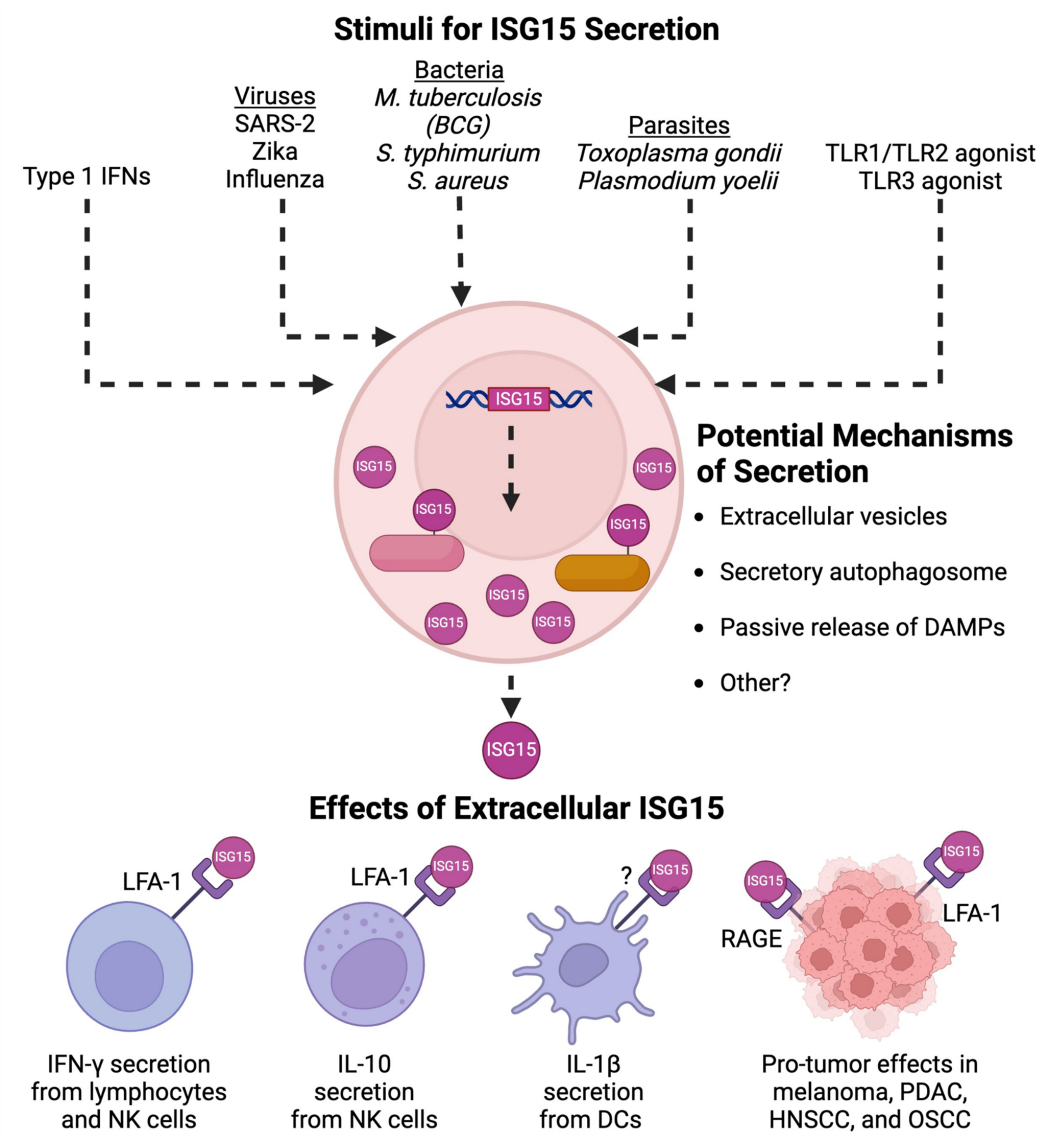
Summary diagram of the stimuli leading to ISG15 secretion, potential mechanisms of ISG15 secretion, and the known functions of extracellular ISG15

Interferon-stimulated genes (ISGs) form the frontline defense mechanism of cells, enabling a rapid response to infection. These genes encode proteins that initiate and amplify the cellular response against invading pathogens. Among the hundreds of ISGs, interferon-stimulated gene 15 (ISG15), a 15 kDa ubiquitin-like molecule, is highly expressed in response to interferon stimulation [1]. The most notable function of ISG15 is that it can be covalently conjugated to target proteins through a process called ISGylation. ISGylated proteins play important roles in viral infection and cancer, and this role of ISGylation has been comprehensively reviewed elsewhere [2–5]. Importantly, numerous studies have found that ISG15 can also be released by cells, particularly during viral infection, yet much less is known about the function of this “extracellular ISG15”. For instance, extracellular ISG15 has been found to be highly secreted after SARS-CoV-2 infection in in vitro models and identified as one of the most highly expressed proteins detected in the serum and plasma of COVID-19 patients [6, 7]. Despite this observation, the role of extracellular ISG15 at different disease stages during infection remains unclear. Additionally, the mechanism of ISG15 secretion has remained elusive. In this mini-review, we summarize our current understanding of extracellular ISG15, including potential mechanisms of secretion along with its functions (Fig. 1).

### Free vs. non-free ISG15

**Fig. 2:**
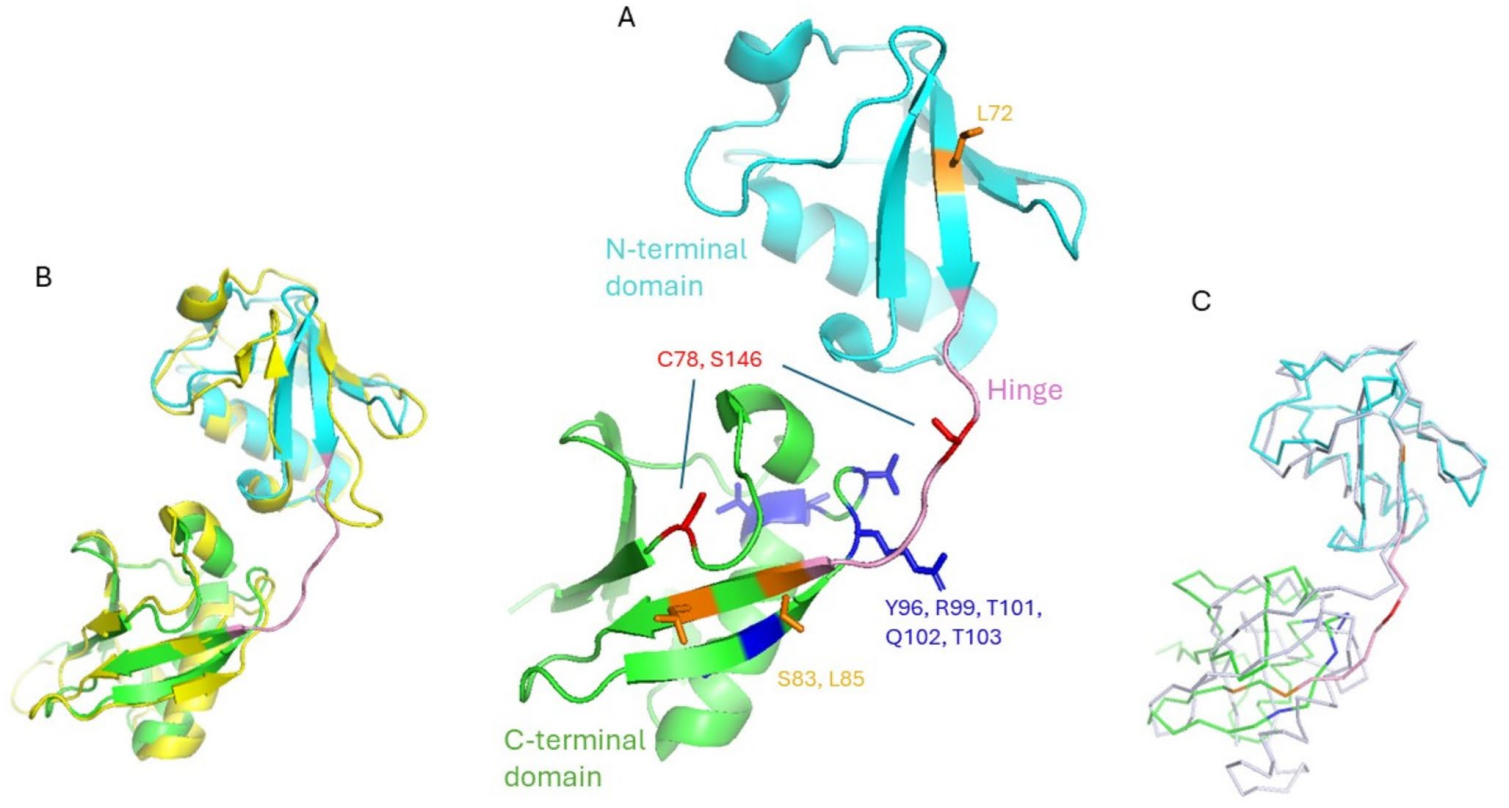
(**A**) Human ISG15 (PDB 1Z2M) with residues important for function highlighted. The molecule comprises an N-terminal domain (Cyan), a hinge region (pink) and a C-terminal domain (green). Two cysteine residues Cys78 and Ser146 (red) were implicated in multimerization. Note that in this published crystal structure Cys78 was intentionally mutated to a serine. Leu72, Ser83 and Leu85 (orange) are implicated in secretion and a bundle of polar residues Tyr96, Arg99, Thr101, Gln102 and Thr103 (blue) are implicated in signaling, putatively interacting with LFA-1. (**B**) Two ubiquitin molecules (extracted from PDB 2K6D, chain B, coloured yellow) are aligned to the two domains of ISG15, showing good structural homology. (**C**) Structural alignment of murine ISG15 (ribbon in light purple) onto human ISG15. While the N-termini are aligned in good agreement, the C-termini adopt different relative orientations to the N-termini due to flexibility of the hinge region

ISG15 is a ubiquitin-like (Ubl) modifier protein that becomes functional after activation through protease cleavage at its LRLRGG motif near the C-terminus [8, 9]. The uncleaved form of human ISG15 is 165 amino acids long, with a high sequence homology to ubiquitin. The hexapeptide cleavage site is highly conserved across species and is also present in ubiquitin [4, 10]. Crystal structures of human ISG15 reveal that a monomeric ISG15 molecule consists of N-terminal and C-terminal domains, both adopting a β-grasp fold—a characteristic also found in ubiquitin [9, 11]. The root-mean-square deviations (RMSDs) for the N-terminal and C-terminal domains compared to ubiquitin are 1.7 Å and 1.0 Å, respectively [9], highlighting their significant structural homology with ubiquitin. In ISG15, the two domains are connected by a flexible hinge region spanning residues Val75 and Leu82, making ISG15 resemble a di-ubiquitin molecule (Fig. 2). This structural similarity suggests that ISG15 may share functional properties with ubiquitin [4, 11–14].

The most well-defined function of ISG15 is its ability to conjugate to target proteins within the cell through ISGylation, which mediates downstream signaling cascades [3, 15]. However, ISGylation may not be the only function of ISG15. An alternative role for ISG15 during viral infection has been demonstrated in knockout mouse models. For instance, a higher survival rate was observed in Ube1L⁻/⁻ mice compared to ISG15-/- mice following Chikungunya virus infection [16]. Similar observations were also seen in influenza A virus but not Sendai virus infection in mice [17]. These findings suggest an antiviral role for “free” ISG15, though the exact mechanism remains unclear.

### Stimuli for releasing extracellular ISG15

It is well-established that type I interferon (IFN) is the primary stimulus for triggering the expression of ISG15 [18, 19]. Knight Jr. and Cordova first demonstrated that primary human lymphocytes and monocytes could release ISG15 into the supernatant following IFN-β treatment [20]. Further studies revealed that ISG15 could also be released from various epithelial cell lines after stimulation of IFN-β [21]. Interestingly, significant upregulation of ISG15 was detected in the serum of human volunteers after administration of IFN-β or during infection [21, 22]. In addition to type I interferon, toll-like receptor ligands such as poly IC and PAM3CSK4, as well as live or inactivated pathogens, have been shown to stimulate ISG15 secretion [13]. Notably, some stimuli induce even higher level of ISG15 secretion than IFN alone. For example, leukocytes stimulated with BCG secrete more ISG15 than those stimulated with IFN-α [23]. Moreover, human macrophages infected with SARS-CoV-2, Zika virus, and influenza all release more ISG15 compared to IFN-β stimulation alone [22].

Interestingly, ISG15 secretion can be mediated through both IFN-dependent and IFN-independent pathways, as demonstrated using IFNAR1-/- cells along with a protein that blocks type I IFN signaling [13]. Furthermore, certain cells secrete ISG15 without any exogenous stimulus under specific biological conditions. For instance, EBV-immortalized B cells (EBV-B cells) and SV-40-immortalized fibroblasts (SV-40 cells) constitutively secrete ISG15 in the absence of any noxious stimulus [23]. Additionally, some tumor cells, such as human melanoma cells and nasopharyngeal carcinoma (NPC) cells [24, 25], are highly active in secreting ISG15. Stimuli for ISG15 secretion are summarized in Table 1.

**Table 1:** Stimuli that trigger ISG15 secretion

Stimulus	Target/Model System	Detection Method	Ref.
Type I IFNs	Human lymphocytes and monocytes	Western blot	[20]
	THP-1 cells	Western blot	[20, 21]
	Human PBMCs Human CD3+, CD4+, and CD8 + cells Human epithelial cells (OVCAR3 & A549) Raji cells Jurkat cells	Western blot	[21]
	Human volunteer serum	ELISA	[21]
	Primary human NK cells NK-92 cells	IFN-γ reporter assay	[13]
***Viral Infection***			
SARS-CoV-2	COVID-19 patient serum	ELISA	[22]
SARS-CoV-2 Zika virus Influenza	Human macrophages		
Poly(I: C) (TLR3 agonist)	Human PBMCs	ELISA IFN-γ reporter assay	[13]
	Mouse splenocytes	IFN-γ reporter assay	
***Bacterial Infection***			
*M. tuberculosis* (heat killed)	Human PBMCs	ELISA IFN-γ reporter assay	[13]
	Mouse splenocytes Primary human NK cells Primary human T cells	IFN-γ reporter assay	
*S. typhimurium* (heat killed)	Human PBMCs	ELISA IFN-γ reporter assay	
	Mouse splenocytes	IFN-γ reporter assay	
*S. aureus* (heat killed)	Human leukocytes	Western blot	[23]
BCG	Human leukocytes	Western blot	
	Human PBMCs	IFN-γ reporter assay	[13]
Pam3CSK4 (TLR1/TLR2 agonist)	Human PBMCs	ELISA IFN-γ reporter assay	
	Mouse splenocytes	IFN-γ reporter assay	
***Parasitic Infection***			
*Toxoplasma gondii* (live)	C57BL/6 mice	ELISA Western blot	[44]
*Plasmodium yoelii* (live)	C57BL/6 mice or ddY mice	Western blot	[53]
***No stimuli***			
None	EBV-immortalized B cells (human) SV-40-immortalized fibroblasts (human)	Western blot	[23]
	ISG15-expressing HEK293Ts	Western blot	[13, 23]
	ISG15-expressing Jurkat cells	IFN-γ reporter assay	[13]
	Melanoma cells (human)	ELISA	[24]
	NPC cells (human)	Western blot (IP)	[25]

### Factors contributing to the release of extracellular ISG15

The relationship between ISGylation and ISG15 secretion remains unclear, but it is plausible that these processes may compete for the available ISG15. Experiments using siRNA to knock down the expression of downstream enzymes UBE1L or HERC5 showed an increase in extracellular ISG15 after Zika or SARS-CoV-2 infection [22]. Conversely, promoting ISGylation by adding E1, E2, and E3 enzymes in ISG15-expressing cells reduced extracellular ISG15 secretion [13]. Importantly, three key residues on ISG15 were identified that regulate its secretion under similar experimental conditions [13]. Beyond intrinsic factors, the interaction between ISG15 and viral proteins also influences the amount of ISG15 released extracellularly. For instance, the NS1B protein of influenza B virus binds non-covalently to both free and conjugated forms of ISG15, leading to reduced ISGylation and secretion [26, 27]. On the other hand, viral proteases such as PLpro of SARS-CoV-2, the OTU domain protease of Nairovirus, and Lbpro protease of picornavirus exhibit strong deISGylase activity, enhancing the extracellular signaling function of ISG15 [13].

Typically, proteins with signal peptides are synthesized in the endoplasmic reticulum and trafficked in COPII-coated vesicles to the Golgi apparatus for subsequent exocytosis via the classical secretory pathway [28]. However, some proteins are secreted through unconventional pathways [29]. Since ISG15 lacks a secretory signal, its release mechanism into the extracellular space remains unclear. Multiple mechanisms have been proposed, including secretion via granules [23], microvesicles [30], and exosomes [31, 32]. Recent studies have investigated ISG15 secretion pathway in the context of viral infection [22]. The secretion was shown to be insensitive to Brefeldin A, confirming that ISG15 does not follow the conventional pathway [22]. However results suggest that the secretion is likely linked to the secretory autophagy pathway, which is an unconventional secretion process where cellular content or viral particles are sequestered by autophagosomes and then secreted to the extracellular space by autophagy and endosomal regulators [33]. Further evidence also suggests that ISG15 secretion is linked to secretory autophagosomes during SARS-CoV-2 infection, as knockdown of components of the secretory autophagy pathway, including ULK1, Lyn kinase, STX3/STX4, or SNARE, abolished ISG15 release [22]. However, the exact secretion mechanism may vary under different conditions. Moreover, ISG15 – like other damage associated patterns (DAMPS) - can also be released passively as an alarmin through inflammatory cell death as has been previously reported [34]. Notably, ubiquitin has also been found in the extracellular space, which is secreted by both passive and active release mechanisms [11, 14]. Therefore, the detailed mechanism of ISG15 secretion requires further investigation.

### Functions and mechanisms of extracellular ISG15

**Fig. 3:**
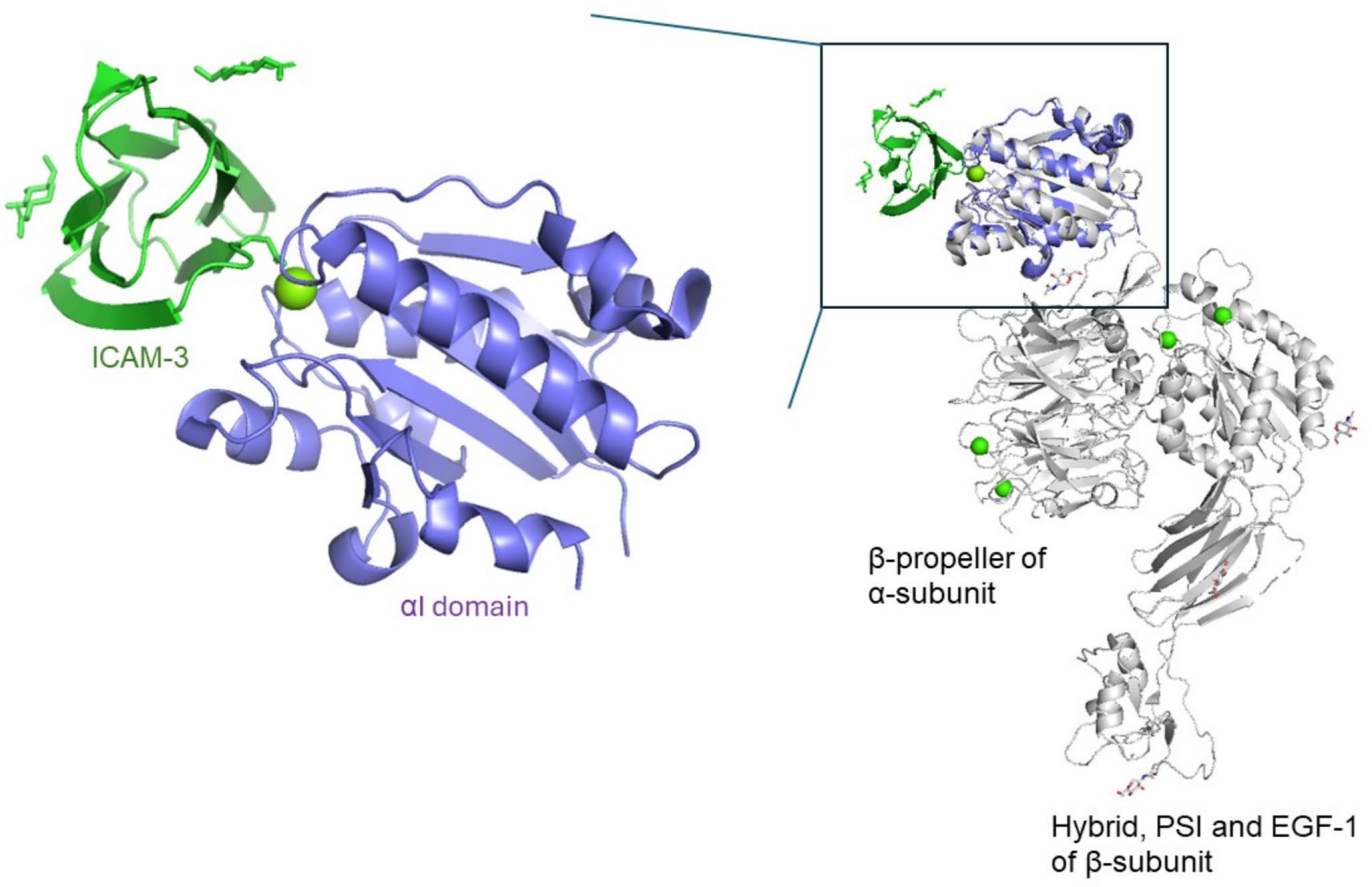
Structure of part of the LFA-1 ectodomain (PDB 5E6R). Enlarged from the inlet shows the aligned structure of the ICAM-3/LFA-1 αI complex (PDB 1T0P). The two molecules are linked up by a magnesium (II) ion. However ISG15 is expected to bind to a different, previously uncharacterized site on this domain and the binding is metal-independent

For extracellular ISG15 to exert its cytokine function, it must bind to a cell surface receptor to activate downstream signaling. Host integrin αLβ2 (also known as lymphocyte function-associated antigen-1, LFA-1) has been recently identified as the cell surface receptor for extracellular ISG15 [35]. LFA-1 is one of the 24 integrins and is expressed on NK cells, macrophages, and various leukocytes [36]. Its most well-known function is serving as a docking site for intercellular adhesion molecules (ICAMs), which are expressed on a variety of leukocytes, endothelial, and epithelial cells [37–39]. This interaction is crucial for cell-mediated immunity, recruitment of leukocytes to inflammation sites, bidirectional transmission of signals, and regulation of gene expression. LFA-1 is a multi-domain heterodimeric complex with two subunits, α and β, bound via non-covalent interactions [36]. Although the full structure of LFA-1 is not yet resolved, the crystal structure of the LFA-1 ectodomain, including the αI and propeller domains of the α subunit and βI, hybrid, PSI, and EGF-1 modules of the β subunit, has been elucidated [40]. The αI domain is the major ligand and inhibitor binding site [41, 42]. For example, ICAM-3 binds to LFA-1 via a metal ion-dependent adhesion site, where a magnesium (II) ion bridges the αI domain and Glu37 of ICAM-3, supported by four surrounding hydrogen bonds (Fig. 3) [43]. ISG15 does not compete with ICAM-1 for binding to LFA-1, suggesting that the two proteins interact with LFA-1 through different binding sites [35]. Four groups of ISG15 residues (Arg99, Thr101, Tyr96, Gln102, Thr103) are important for signaling and are likely involved in receptor binding [35]. While the structure of the ISG15/LFA-1 complex has not yet been solved, several questions remain. For instance, how LFA-1 distinguishes ISG15 from a di-ubiquitin molecule? While Mendoza-Salazar et al. proposed that ubiquitin may lack important amino acids for receptor binding [11], more experimental evidence is needed to support this hypothesis. In addition, it is still unknown if the same outside-in signaling mechanism used by ICAMs also applied to extracellular ISG15? It is important to note that four other proteins—MYO1G, ESYT1, MCTP2, and OGFR—were identified as potential ISG15 receptors using the ISG15 UBAIT (Ubiquitin Activated Interaction Trap) approach [35]. Further investigation is needed to determine whether these proteins can serve as cell surface receptors for extracellular ISG15.

Although the molecular mechanism of extracellular ISG15 remains elusive, the multimerization of secreted ISG15 appears to be necessary for inducing IFN-γ and IL-1β in Toxoplasma-infected mice [44]. In mice, two cysteine residues (Cys76 at the hinge region and Cys144) are important for this multimerization. These cysteine residues can be nitrosylated, which prevents ISG15 dimerization [45] and constitutes a putative regulatory mechanism of secreted ISG15 in mice. However, only the first cysteine of these two is present in human ISG15 (Cys78), with the second one replaced by a serine (Ser146) (Fig. 2) [45]. The mutation from Cys to Ser at this position was found to enhance intracellular ISGylation activity as well [45]. Through analyzing the structure of human ISG15 (PDB 1Z2M) [9], Ser146 is involved in multiple polar interactions with neighboring residues. Such interactions would not be possible if it were a cysteine. These interactions may affect the structural flexibility of the C-terminal strand of the protein. More experimental investigation is worthed to confirm whether this constitutes another regulatory mechanism in human by affecting ISG15 activation at the extreme C-terminus. While it has been shown that binding of viral proteins to the hinge region affects intracellular ISG15 activity [46], residues such as Leu72, Ser83, and Leu85 flanking the hinge region are important for secretion but not receptor binding [13]. Dzimianski et al. suggested that although the tertiary structures of Ubl domains are conserved across species, the interspecies domain orientation varies [10]. Such differences also exist between mouse and human ISG15 where a twist in the mouse C-terminal domain was noted [47]. While Leu72, Ser83, and Leu85 do not directly interact with a SARS-CoV papain-like protease [47], it is possible that these residues affect hinge flexibility and thus C-terminal domain orientation, thereby affecting protease binding and modulating ISG15 activation at the C-terminus indirectly. Further research is needed to explore how sequence diversity and structural flexibility of the hinge region affect extracellular ISG15 functions.

Initial studies on the function of extracellular ISG15 found that priming PBMCs with recombinant ISG15 enhanced LPS-induced monocyte cytotoxicity in a dose-dependent manner [48]. This study also reported that ISG15 treatment stimulated IFN-γ secretion from PBMCs and CD3 + T cells [48]. Additional studies have confirmed the role of extracellular ISG15 in stimulating IFN-γ production from lymphocytes [49]. Another study showed that mouse splenocytes secrete IFN-γ in response to co-stimulation with extracellular ISG15 and IL-12 [35]. In a study on Mendelian susceptibility to mycobacterial disease (MSMD), Bogunovic et al. identified patients with a genetic loss of ISG15 who could not produce IFN-γ in response to the Mycobacterium bovis Bacille Calmette-Guérin (BCG) vaccine, leading to severe disease [23]. Beyond IFN-γ, several studies have shown that extracellular ISG15 also stimulates the secretion of other cytokines. For example, co-stimulation of ISG15 and IL-12 induces the production of IFN-γ and IL-10 in a human NK cell line [35]. Additionally, injecting recombinant ISG15 into Toxoplasma gondii-infected mice increased IL-1β production from CD8α + dendritic cells (DCs) [44].

Other immunomodulatory roles for ISG15 have also been reported. ISG15 has been shown to induce NK-cell proliferation and enhance lymphokine-activated killer-like activity [49]. Furthermore, treating human PBMC-derived macrophages with recombinant ISG15 promotes the cells’ switch to an M2 phenotype [25]. More work is needed to identify the effect of ISG15 on other immune cells.

Extracellular ISG15 has been shown to have activity in cancer as well. For instance, in the context of melanoma, extracellular ISG15 produced by melanoma cells can induce the expression of E-cadherins on human dendritic cells, impairing its mobility and potentially allowing for tumor immune escape [24]. In the context of pancreatic ductal adenocarcinoma (PDAC), extracellular ISG15 was found to be secreted by tumor-associated macrophages which enhanced cancer stem cell phenotypes in PDAC and promoted tumor growth in mice [50]. Additionally, ISG15 released by necroptotic head and neck squamous cell carcinoma (HNSCC) cells can reprogram the microenvironment to enhance tumor progression and metastasis [34]. Importantly, this effect was determined to be through the receptor RAGE, indicating that extracellular ISG15 can mediate its effects through multiple receptors and signaling cascades [34]. Finally, experiments in oral squamous cell carcinoma (OSCC) revealed that extracellular ISG15 produced from OSCC cells promote changes in tumor microenvironment by inducing fibroblasts recruitment, potentially having an effect in cancer progression [51]. Together, these cancer studies highlight the need for studying the role of extracellular ISG15 in cancer. Importantly, given its role in enhancing the growth and invasion of several different cancers, circulating ISG15 may represent both a crucial biomarker and target for therapeutic intervention in different cancers. Taken together, these data establish extracellular ISG15 as a multifunctional cytokine with pleiotropic effects on immune cells and cancer cells in infectious and cancerous contexts.

## Prospects and conclusion

For researchers investigating the potential functions of secreted ISG15, it is crucial to recognize that ISG15 alone may not be sufficient to drive certain phenotypic outcomes. Early studies revealed that ISG15-induced augmentation of monocyte-mediated cytotoxicity does not occur with purified monocytes alone; instead, the presence of other cell populations is necessary [48]. Similarly, the stimulation of NK cell proliferation by ISG15 indicates the requirement for intermediary signals [49]. Subsequent research identified IL-12 as one such intermediary factor, at least in the context of IFN-γ secretion. For example, Bogunovic et al. demonstrated that IL-12 and ISG15 synergistically upregulate IFN-γ production from PBMCs more effectively than either molecule alone [23]. Further, Swaim et al. elucidated that this synergy involves IL-12-induced IFN-γ transcription and ISG15-driven IFN-γ secretion [35]. These findings suggest that extracellular ISG15 may rely on IL-12 or other factors to mediate some of its paracrine functions. Given the variety of infectious stimuli that can induce greater ISG15 release than type I IFN alone, it is plausible that certain viral or bacterial proteins enhance ISG15 secretion from infected cells. One subset of these proteins may promote the generation of free, intracellular ISG15, such as viral deISGylases [52]. The accumulation of free ISG15 through deISGylase activity could provide a larger pool for eventual secretion.

The landmark study on patients with Mendelian susceptibility to mycobacterial disease (MSMD) revealed a critical role for free, extracellular ISG15 in controlling Mycobacterial infections through the stimulation of IFN-γ production [23]. Given that MSMD patients are also more susceptible to Salmonella infections, extracellular ISG15 may similarly be involved in controlling Salmonella, providing another valuable model for studying extracellular ISG15 in bacterial infections. Detailed studies of the stimuli that lead to ISG15 release could identify proteins that modulate extracellular ISG15 levels, presenting promising targets for therapeutic intervention. The questions remain to be addressed are listed in Table 2.

**Table 2:** Questions remaining to be addressed

What co-stimuli are needed for extracellular ISG15 to function?
Does extracellular ISG15 function differently in mice compared to humans?
What viral, bacterial, and parasitic factors are involved in promoting the release of ISG15?
Does extracellular ISG15 have a pro-viral or anti-viral role in the context of viral infection?
Are there other receptors other than LFA-1 that are activated by ISG15?
What other disease model systems can be exploited to study the functions of extracellular ISG15?
How do immortalized/cancerous cells constitutively secrete ISG15?

Over the past few decades, significant advancements have been made in understanding ISG15 and its diverse functions both intracellularly and extracellularly. ISG15 is an immunomodulatory protein secreted by various mammalian cells in response to a wide range of viral, bacterial, and endogenous stimuli. Its elevated levels in the serum of COVID-19 patients underscore a potential role for ISG15 in either combating or exacerbating viral diseases. This gap in knowledge highlights the necessity for further studies into the functions of extracellular ISG15 and the mechanisms governing its secretion. Enhanced understanding of extracellular ISG15 could facilitate the discovery of novel diagnostic and therapeutic strategies for infectious, cancerous, and inflammatory diseases. 

## Data Availability

Not applicable.
